# Supplementary material for: Microplastics inhibit macrophage bioenergetics impairing homeostatic function and immune responsiveness
Source: bioRxiv. 2026 May 18:2026.05.14.725255. Preprint. [Version 1] doi: 10.64898/2026.05.14.725255 (PMC13228642; doi:10.64898/2026.05.14.725255)
Supplement: Supplement 1 — Supplemental Figure 1: Total particle volume per cell represents a consistent metric for administering MNPs in vitro. 20,000 Raw264.7 cells were co-cultured overnight with 1μm (left) or 4μm MNPs (right) at increasing MNP per cell ratios. Total culture volumes were 200μl, 500μl, and 800μl. ATP production was measured by CellTiter-Glo 2 per manufacturers specifications. No significant change in ATP production was observed. Supplemental Figure 2: Confocal imaging of MNP-laden monocyte-derived macrophages. Human MDMs treated with MNPs at 10:1 or 100:1 were cultured overnight. Cells were immunostained with Tom20-specific antibody (red), phalloidin (orange), and DAPI (blue). A) Representative illustration of Tom20 expression seen wrapping around intracellular 4μm MNPs. Supplemental Figure 3: Representative immunofluorescent images showing labeled MNPs in the lung, brain, liver, kidney, colon, heart and spleen. Lung image is of a PCLS generated from mouse tissue 4hrs post-administration of 1μm MNPs (pseudo-color of 3 different fluorescent emissions). Supplemental Table 1: Details of MNP dosage per experiment reported as particles per cell, concentration (conc.), and particle volume per cell. Supplemental Tabel 2: List of conventional macrophage-specific transcripts used to compare the effects of MNPs, DEP, and saline following in vitro administration to MDMs. [file media-1.pdf]

Supplemental Data – Extended Methods

Polystyrene MNPs and uptake: Polystyrene microspheres of 1μm, 4μm, and 9μm labeled with either yellow-green (excitation/emission maxima of 505/515 nm), red (580/605 nm), or crimson (625/645 nm) were purchased from ThermoFisher Scientific. Non-fluorescent, unlabeled polystyrene microspheres of 1μm, 4μm, and 9μm were purchased from Spherotech. Human monocyte-derived macrophages (MDM) were generated by culturing magnetic bead-isolated $CD14^+$ cells from donor blood (N = 6, Vitalant) in 20 ng/ml M-CSF for 5-6 days. MDMs were cultured in RPMI-1640 supplemented with 10% heat-inactivated fetal bovine serum (FBS), 2 mM L-glutamine, and 10 U/mL Penicillin-Streptomycin (P/S). $5x10^4$ MDMs were seeded overnight in 4-well Lab-Tek II chamber slides and allowed to adhere at 37°C, 5% CO_2 followed by co-culture with MNPs for 24 hours. Cells were then fixed and permeabilized using the True-Nuclear Transcription Factor Buffer Set (BioLegend). Cells were stained with Rab5a antibody (Alexa647; Santa Cruz sc-46692), Rab7 antibody (Alexa647; Santa Cruz, sc-376362), and LAMP-1 antibody (Alexa647; Santa Cruz, sc-19992) in permeabilization buffer overnight. Nuclei were stained with DAPI and the microfilaments were stained with Phalloidin (Alexa488; cell signaling, 8878S). The fluorescence was photographed by an EVOS M5000 microscope. 20,000 Raw264.7 murine macrophages (TIB-71, ATCC) were seeded in 100 μl DMEM supplemented with 10% FBS, 2 mM L-glutamine, and 10 U/mL P/S into 96-well flat-bottom plates and incubated for 4h at 37°C, 5% CO_2 to allow for adhesion. Cells were exposed to microplastics at varying concentrations for 24 h. Prior to analysis, cells were washed 3 times to remove any unbound MNPs. MNP uptake was measured via immunofluorescent imaging on an

EVOS M5000 microscope. All conditions were performed in triplicate and experiments were repeated a minimum of three times independently.

LDH release: 20,000 Raw264.7 cells (ATCC, TIB-71) were co-cultured with MNPs in a final volume of 100 µl with complete DMEM for 24 hours. Lactate dehydrogenase release was quantified using the CyQUANT™ LDH Cytotoxicity Assay Kit (Invitrogen) according to the manufacturer's instructions. Cell-free supernatants (50µL) were collected in a 96-well plate, mixed with 50µL reaction mixture, and incubated for 30 min at room temperature in the dark. 50ul of stop solution was added and absorbance was read at 490 nm and 650 nm (background) using a Varioskan ALF microplate reader. Percent cytotoxicity was calculated as: (Sample−Spontaneous LDH activity)/(Maximum LDH activity−Spontaneous LDH activity)×100 using background-corrected values ($A_{490} - A_{650}$).

XTT assay: For viability, metabolic activity was measured using the CyQUANT XTT Cell Viability Assay Kit (Invitrogen). A working solution (1:6 electron coupling:XTT reagent) was prepared immediately before use, and 70µl was added to each well. After 4h at 37°C, absorbance was recorded at 450 nm and 650 nm using a Varioskan ALF microplate reader. Specific absorbance was calculated as [Abs_{450} nm(Test) – Abs_{450} nm(Blank)] – Abs_{650} nm(Test)], and viability was expressed relative to untreated controls.

Transcription factor activation: $2x10^5$ Raw-Dual reporter cells (Raw264.7 dual NF-κB/IRF reporter cells; InvivoGen, rawd-ismip) were seeded in 200 µl in 96-well plates and exposed to increasing concentrations of diesel exhaust particles (25-400 µg/mL), 1µm MNPs (100:1-1600:1,

MNPs/cell), or 4μm MNPs (10:1-160:1, MNPs/cell) with or without 100μg lipopolysaccharide (LPS) for 24hrs. NF-κB activation was measured by the secreted embryonic alkaline phosphatase (SEAP) reporter using QUANTI-Blue detection reagent (InvivoGen) according to the manufacturer's instructions and absorbance measured at 620nm. Interferon response factor (IRF) activation was determined using the Lucia luciferase reporter and quantified using QUANTI-Luc detection reagent (InvivoGen) following the manufacturer's flash luminescence protocol and immediately read on a Varioskan ALF microplate reader. Reporter activity was normalized to untreated cells (LPS- conditions) or LPS-treated controls (LPS+ conditions), and results were expressed as percentage changed relative to the corresponding control.

JC-1 Assay: 5x10^4 Raw264.7 cells were seeded in Lab-Tek II Chamber Slides and co-cultured in 1ml DMEM with various concentrations of 4μm microplastics for 24-hours at 37°C. CCCP (carbonyl cyanide 3-chlorophenylhydrazone) was used as a positive control (ThermoFisher; 2μM). Cells were then stained with 2μM JC-1 reagent (MitoProbe, ThermoFisher) at 37°C in dark for 30 minutes. Cells were washed thoroughly, labeled with DAPI, and imaged for immunofluorescence. Three-dimensional (3D) fluorescence z-stacks were acquired to characterize mitochondrial activation profiles around the microplastics. Microplastic centroids in (x, y, z) were identified by combining their detection within each 2D optical z-section using a Hough circle transform with their reconciliation in 3D using 3D anisotropic DBSCAN algorithm. For each detected microplastic, radial intensity profiles were extracted in the green and red channels, representing mitochondrial activation and inactivation states, by computing the 3D Euclidean distance from the centroid to every surrounding voxel. Following per-slice background subtraction and per-channel peak normalization, profiles were averaged across all

microplastics to characterize the spatial distribution of mitochondrial activation with respect to the microplastics.

ROS Assay: 5x10^5 Raw264.7 cells in 1ml DMEM were co-cultured with 1µm or 4µm MNPs at increasing MNP/cell ratios for 24-hours. Cells were then labeled with 500nM CellROX green kit for 30-60 min at 37°C with 5µm Sytox red included for the final 15 minutes (ThermoFisher). TBHP (Tert-butyl hydroperoxide) (400µm) was added for 1 hour at 37°C as a positive control. JC-1 was immediately measured on a CytoFLEX cytometer and reported as red/green ratio (Beckman Coulter).

Cytokine Production: 50µl of fresh cell culture supernatant was analyzed using the Mouse Inflammation Cytometric Bead Array kit simultaneously measuring IFNγ, TNFα, IL-6, IL-10, CCL2, and IL-12p70 (BD Biosciences). Data was collected using a CytoFLEX flow Cytometer and analyzed using FlowJo V10 software (BD Biosciences). Cytokine levels were determined per mean fluorescence intensity measurements from manufacturer provided standards and protocol using GraphPad Prism8 (GraphPad Software).

Mitochondrial Mass Assay: Mitochondrial mass was assessed using Tom20 (1:400, CST, 42406) co-stained with Alexa 647 (1:100, Jackson Immuno, 111-605-003). Briefly, cells were fixed in 3.7% PFA in PBS for 5 minutes at 37°C. The cells were permeabilized using 0.25% Triton X-100 in PBS, blocked with 10% goat serum in PBS. Cells were then stained with Rhodamine Phalloidin following manufacturer's protocol (Fisher Scientific, R415) and DAPI. Images were then obtained by confocal microscopy using a 60x objective and Olympus IX83 microscope

equipped with a Cicero widefield/confocal system. Analysis was performed using ImageJ and CellProfiler software.

Seahorse XF Mito Stress Test: Mitochondrial respiration was measured using the Seahorse XF Mito Stress Test Kit (Agilent Technologies,103010-100) according to the manufacturer's instructions. Briefly, cells were seeded in Seahorse cell culture microplates at a density of 100,000 cells and incubated overnight to allow adherence with or without 100:1 4µm MNPs or 50 µg/ml DEP. Prior to the assay, culture medium was replaced with Seahorse XF Assay Medium (Agilent) supplemented with 10 mM glucose, 2 mM glutamine, and 1 mM pyruvate, and cells were incubated in a non-CO_2 incubator at 37°C for 1 hour. Oxygen consumption rate (OCR) was measured under basal conditions and following sequential injections of oligomycin (1 µM), FCCP (1 µM), and rotenone/antimycin A (0.5 µM each). Data were normalized to total cell count per well and analyzed using Wave software (Agilent).

Phagocytosis: Raw264.7 cells were co-cultured overnight with 10:1 4µm MNPs then incubated with 5:1 Zymosan A (S. cerevisiae) BioParticles per cell (5:1, ThermoFisher) for 4 hours. Some groups were pre-treated for 1 hours with AICAR (10-100 µM) prior to zymosan addition. Zymosan uptake was measured as the percent of cells expressing AlexaFluor488 by flow cytometry.

OVA uptake and stimulation assay: 2.5x10^5 Raw264.7 cells were seeded in sterile chambered glass slides (Lab-Tek™ II Chamber Slide™ System, ThermoFisher) and co-cultured with 4µm red MNPs (580/605 nm) at 10:1 overnight. Cells were washed three times then cultured with

FITC-labeled ovalbumin protein (25μg/ml; Invitrogen) for 2-4 hours at 37°C. Cells were washed, fixed, permeabilized then stained with LAMP-1 (1:100) at room temperature for 90 minutes followed by extensive washing then DAPI for 5 minutes and visualized via immunofluorescence imaging. $5x10^5$ Raw264.7 cells were seeded in 12-well plates (2ml DMEM) and cultured overnight (16-18hrs) with (100U/ml) IFNγ with or without microplastics. Unbound MNPs were removed, cells washed, then cultured with ovalbumin (OVA) protein (Sigma; 100μg/mL) or $OVA_{323–339}$ peptide (Sigma; 20μg/mL) for 4h at 37°C. DO11.10 T cells (Cat# 85082301, MilliporeSigma) were maintained in culture with complete DMEM at 37°C, 5% CO_2. DO11.10 cells were labeled with 0.5μM CFSE (Invitrogen) for 10 minutes at room temperature. CFSE was quenched by adding 4-5 volumes of cold media and incubated on ice for 5 minutes then washed 3 times with complete media. CFSE-labeled DO11.10 cells were added to the OVA protein or peptide treated Raw264.7 cells at a 1:1 ratio in fresh DMEM medium. Co-cultures were maintained at 37°C in 5% CO_2 incubator for 72h. Non-adherent DO11.10 cells were then collected, stained for viability with zombie NIR (1:1000; BioLegend) at 4°C in dark for 15 minutes, and immediately analyzed via flow cytometry. Checkpoint inhibitors anti-PD-1 (BioXcell, BP0146) and anti-PD-L1 (BE0101) were included at 30μg/ml in some experiments. Proliferation was normalized to $CSFE^{Low/null}$ cells stimulated in the absence of MNPs. Data was analyzed using FlowJo software (v10.10).

In vivo exposures: 10- to 16-week-old female and male FVB/N mice (Jackson Laboratory) were maintained in a specific pathogen-free environment at the University of Pittsburgh animal research facilities at the UPMC Hillman Cancer Center. Mice were administered $3x10^7$ 1μm MNPs equal parts yellow-green, red, and crimson in sterile saline or sterile saline alone

intranasally in 40µl administering 20µl/nare. At 4-hours, 1-day, or 1-week post-administration mice were euthanized and lung, liver, heart, kidney, brain, colon, and spleens were harvested and processed into single cell suspension with mechanical digestion only. Red blood cells were lysed from blood samples using BD Pharm Lyse Buffer (Waters Biosciences). Single cell suspensions were immediately analyzed via flow cytometry for detection of 505/515nm, 580/605nm, or 625/645nm fluorescence. Data was analyzed in FlowJo v10.10 and fluorescent MNP counts normalized to 10^6 total events. Additional mice received non-fluorescent MNPs at $3x10^7$ 1µm or $5x10^5$ 4µm MNPs, or sterile saline intranasally. 1-week post-administration, lungs were excised with thymus and heart carefully removed, finely minced, and digested at 37°C, 5% CO_2 for 30 min in the presence of Liberase TM (Millipore Sigma) per manufacturer's instructions. Mechanically and enzymatically dissociated lung homogenate was pressed through a 100µm filter with the plunger of a 5ml syringe followed by extensive washing with cold, sterile PBS. Lung homogenate was then passed through a 70µm filter and stained for CD45, CD11b, CD88, CD24, CD64, Ly-6G, then LiveDead blue viability dye. Pulmonary macrophages were isolated as $LiveDead^-$ $CD45^+$ $CD11b^+$ $CD64^+$ $CD88^+$ $CD24^-$ $Ly\text{-}6g^-$ cells to ≥96% purity with a Sony900 cell sorter. All experiments were approved by the University of Pittsburgh Institutional Animal Care and Use Committee and conducted in accordance with guidelines of the U.S. Public Health Service Policy on Humane Care and Use of Laboratory Animals.

Human lung tissue procurement: The use of human lung tissue was approved by the University of Pittsburgh Institutional Review Board. Lung tissue unsuitable for transplantation was obtained from the Pulmonary, Asthma, Critical Care, and Sleep Medicine Thoracic Tissue Repository and the Center for Organ Recovery and Education. After collection, lung lobes were stored on ice in

DMEM/F12 (Dulbecco's Modified Eagle Medium and Ham's F 12 Nutrient Mixture) or in an organ transplantation preservation solution until processing within 3 hours of receipt. All experimental procedures were performed under sterile conditions.

Human precision cut lung slice (hPCLS): hPCLS were generated as previously described[27]. Briefly, lung lobes were inflated via the main bronchus with 500–600 mL of warm (37–40 °C) 2.5% low–melting point agarose (LMPA; IBI Scientific) prepared in DMEM/F12 with phenol red (ThermoFisher, Waltham, MA) and supplemented with 1% fetal bovine serum (FBS; BioTechne), 1% penicillin/streptomycin, and 1% amphotericin B. The inflated tissue was then kept on ice for 30-45 minutes to allow agarose to solidify. Agarose filled lobes were then cut into approximately 2.5cm thick slices. Tissue cores were obtained from each slice using a 1cm diameter biopsy coring tool (Alabama Research and Development), avoiding major airways and blood vessels. Parenchymal cores were maintained on ice in DMEM/F12 supplemented with 1% FBS, 1% PenStrep, and 1% Amphotericin B (Complete Media 1%). Precision cut lung slices (300 μm thickness) were generated using a Compresstome vibroslicer (VF 510 0Z, Precisionary Instruments) with a cutting speed of 6μm/s and an oscillation frequency of 6Hz. Individual slices were placed into 24 well plates. Plates were incubated overnight at 37 °C in a humidified 5% CO_2 atmosphere in complete media 1%. The following day, complete media 1% was replaced with DMEM/F12 supplemented with 0.1% FBS (Complete Media 0.1%) and HPCLS were exposed to MNPs, DEP, or saline for 24hrs.

hPCLS cell viability assessment: To evaluate hPCLS viability and survival, two to three 4mm punches were obtained from a single 1cm hPCLS using a biopsy punch. Samples were assessed

in triplicate on prior to and at the end of treatments. The CellTiter-Glo 2.0 ATP quantification assay (Promega) was used according to the manufacturers' instructions. LiveDead viability assay was used including cell-permeant Calcein AM which is converted to green-fluorescent calcein by intracellular esterases indicating metabolically active cells, and the cell-impermeant BOBO-3 Iodide which produces red fluorescence when bound to DNA/nucleic acids inside dead or dying cells.

RNA-sequencing: Murine pMacs were isolated 1 week after MNP administration. Human MDMs were treated in vitro with 4µm MNPs at 100:1, 50µg DEP, or saline for up to 3 days. RNA was extracted using the RNeasy kit (Qiagen). Generation of cDNA, library preparation, and sequencing were performed by the Health Science Sequencing Core at UPMC Children's Hospital and analysis provided by the Biostatistics Facility of the UPMC Hillman Cancer Center.

Statistical analysis: Data between groups were analyzed using non-parametric Kruskal-Wallis test followed by Bonferroni-adjusted p-values for post-hoc comparisons using and GraphPad Prism11 (GraphPad Software). All in vitro assays were performed a minimum of 3 times independently. For all hypothesis, statistical significance was denoted as $p \leq 0.05$ (*), $p \leq 0.01$ (**), $p \leq 0.001$ (***), and $p \leq 0.0001$ (****). All data are reported as the mean ± SEM.

Figure	Size	Ratio	Conc. (μg/ml)	PV (μm^3)	PV/cell (μm^3)	Cells/vol
**1D/1E**	1μm	1,000:1	110	1.048E+07	5.240E+02	$2x10^4$/100
	1μm	5,000:1	550	5.240E+07	2.620E+03	
	1μm	10,000:1	1,100	1.048E+08	5.240E+03	
	1μm	50,000:1	5,500	5.240E+08	2.620E+04	
	4μm	100:1	704	1.048E+06	5.240E+01	
	4μm	500:1	3,520	5.240E+06	2.620E+02	
	4μm	1,000:1	7,040	6.702E+08	3.351E+04	
	4μm	5,000:1	35,200	3.351E+09	1.676E+05	
**2A/2B**	1μm	100:1	55	1.048E+07	5.240E+01	$2x10^5$/200
	1μm	200:1	110	2.096E+07	1.048E+02	
	1μm	400:1	220	4.192E+07	2.096E+02	
	1μm	800:1	440	8.384E+07	4.192E+02	
	1μm	1,600:1	880	1.677E+08	8.384E+02	
	4μm	10:1	352	6.702E+07	3.351E+02	
	4μm	20:1	704	1.340E+08	6.702E+02	
	4μm	40:1	1,408	2.681E+08	1.340E+03	
	4μm	80:1	2,816	5.362E+08	2.681E+03	
	4μm	160:1	5,632	1.072E+09	5.362E+03	
**2C/2D**	1μm	10:1	1.1	1.048E+06	5.240E+00	$2x10^5$/1,000
	1μm	100:1	11	1.048E+07	5.240E+01	
	1μm	500:1	55	5.240E+07	2.620E+02	
	1μm	1,000:1	110	1.048E+08	5.240E+02	
	1μm	5,000:1	550	5.240E+08	2.620E+03	
	4μm	10:1	70.4	6.702E+07	3.351E+02	
	4μm	50:1	352	3.351E+08	1.676E+03	
	4μm	100:1	704	6.702E+08	3.351E+03	
	4μm	200:1	1,408	1.340E+09	6.702E+03	
	4μm	500:1	3,520	3.351E+09	1.676E+04	
**3A/3B**	4μm	100:1	1,760	1.676E+08	3.351E+03	$5x10^4$/100
**3C**	4μm	1:1	17.6	1.676E+07	3.351E+01	
	4μm	3:1	52.8	5.027E+07	1.005E+02	
	4μm	6:1	105	1.005E+08	2.011E+02	
	4μm	12:1	211	2.011E+08	4.021E+02	
	4μm	25:1	440	4.189E+08	8.378E+02	
**3E/6C/6E**	1μm	10:1	6.88	1.310E+06	5.240E+00	$2.5x10^5$/200
**3F**	1μm	100:1	6.88	1.310E+06	5.240E+01	$2.5x10^4$/200
	4μm	100:1	440	8.378E+07	3.351E+03	
**3H**	1μm	100:1	110	2.620E+06	5.240E+00	$5x10^5$/250
**4A**	1μm	10:1	0.55	5.240E+05	5.240E+00	$1.0x10^5$/1000
	1μm	100:1	5.5	5.240E+06	5.240E+01	
	1μm	1,000:1	55	5.240E+07	5.240E+02	
	4μm	10:1	35.2	3.351E+07	3.351E+02	
	4μm	100:1	352	3.351E+08	3.351E+03	
	4μm	1,000:1	3520	3.351E+09	3.351E+04	
**4B/4D**	1μm	100:1	0.69	1.310E+07	5.240E+01	$2.5x10^5$/2,000
	1μm	500:1	3.44	6.550E+07	2.620E+02	
	1μm	1,000:1	6.88	1.310E+08	5.240E+02	
	1μm	5,000:1	34.38	6.550E+08	2.620E+03	
	4μm	10:1	44	8.378E+07	3.351E+02	
	4μm	50:1	220	4.189E+08	1.676E+03	
	4μm	100:1	440	8.378E+08	3.351E+03	
	4μm	200:1	880	1.676E+09	6.702E+03	
**4F**	4μm	6:1	26.4	5.027E+07	2.011E+02	$2.5x10^5$/2,000
	4μm	12:1	52.8	1.005E+08	4.021E+02	
	4μm	25:1	110	2.094E+08	8.378E+02	

Gene	DEP vs Ctrl	MNP vs Ctrl	MNP vs DEP
ABCA1	0.126513969	0.781459382	0.098276756
ABCG1	0.487107251	0.275943972	0.018439725
ACTG1	0.728114292	0.149281147	0.017915666
ACTN1	0.501872477	0.006533714	0.006761141
ADAM10	0.492941844	0.739081537	0.641580735
AIF1	0.62067426	0.313984622	0.499142003
ALOX5	0.030165384	0.395212671	0.082482657
APOE	0.445301144	0.015611133	0.02865042
ARF3	0.380455397	0.541400832	0.050719326
ATF3	0.954971095	0.100383312	0.035846228
ATF4	0.013750559	0.008515036	0.831826387
ATG10	0.61309691	0.922474935	0.435414314
ATG101	0.853982057	0.99809598	0.811312871
ATG12	0.90682091	0.282538174	0.214305885
ATG13	0.697699362	0.419085875	0.58704987
ATG14	0.565049181	0.606124273	0.153794021
ATG16L1	0.865131352	0.85081797	0.642590381
ATG3	0.036593721	0.059881416	0.777300351
ATG5	0.281956047	0.048678155	0.250991716
ATG7	0.882936944	0.426621002	0.392945207
ATG9A	0.072864047	0.062204372	0.929651069
ATP5F1B	0.97666377	0.996881301	0.659078548
ATP5PB	0.572180605	0.425619435	0.759541554
ATP6V0B	0.623456025	0.186605374	0.272708392
ATP6V0C	0.079138039	0.012594839	0.328645357
ATP6V0D1	0.659436257	0.49425674	0.137988067
ATP6V0D2	0.05784168	0.824445466	0.026980256
ATP6V0E1	0.751434506	0.006885936	0.001641562
ATP6V1A	0.594490054	0.269262514	0.449284423
B2M	0.64424122	0.000124965	8.08455E-06
BAX	0.434039055	0.765049308	0.526407169
BCL2	0.87925196	0.311873132	0.262338243
BCL2A1	0.918220069	0.438684683	0.246063721
BECN1	0.835122276	0.342416978	0.33605147

BNIP3	0.790742009	0.099804768	0.070590316
BNIP3L	0.881285998	0.166299159	0.042892304
CASP1	0.823695364	0.661699355	0.384256943
CCL18	0.748765434	0.026271118	0.013280744
CCL2	0.779888431	0.923941825	0.619960328
CCL22	0.581829018	0.006678362	0.004238674
CCL23	0.523276797	0.218075153	0.015920876
CCL3	0.939019317	0.042147102	0.005418573
CCL3L1	0.075513078	0.017785541	7.82768E-08
CCL4	0.745699971	0.185482843	0.031719556
CCL4L1	0.167776449	0.68568544	0.019552472
CCL5	0.822550536	0.278137249	0.085553645
CCR1	0.746006531	0.501477038	0.188344114
CCR5	0.227550041	0.657045281	0.030051366
CCRL2	0.610353293	0.78189333	0.299138268
CD14	0.728273034	0.850662227	0.478585363
CD151	0.282569331	0.154733665	0.648746202
CD200R1	0.349990163	0.432957191	0.842014548
CD209	0.846189808	0.905431112	0.679889878
CD22	0.330262067	0.603358657	0.550007103
CD274	0.072299663	0.000966968	0.048700705
CD276	0.609011733	0.052493648	0.059020278
CD28	0.666852327	0.549284382	0.824543424
CD300A	0.937938322	0.020315418	0.003144302
CD300C	0.777312079	0.249572951	0.252365113
CD300LB	0.489667208	0.093619521	0.192850161
CD300LF	0.556646861	0.674877363	0.823865558
CD302	0.717107907	0.500731807	0.681732403
CD33	0.867434244	0.718698628	0.494356897
CD36	0.986532104	0.783137192	0.699852752
CD37	0.457530297	0.801839931	0.189304897
CD40	0.796936623	0.413877583	0.460783322
CD44	0.313369352	0.107090339	0.425252204
CD47	0.537042449	0.482601436	0.911574627
CD48	0.538141509	0.12361005	0.223090882

CD52	0.971776939	0.371750584	0.256476409
CD63	0.723305767	0.017540436	0.007523927
CD68	0.071613278	0.881621273	0.009887785
CD74	0.077853911	0.072415553	0.965040085
CD80	0.987720326	0.630353049	0.517809834
CD81	0.371478624	0.052684313	0.167826541
CD82	0.499575989	0.509258376	0.078838863
CD83	0.740926997	0.142781856	0.133699232
CD84	0.048054046	0.01805406	0.609023853
CD86	0.583064206	0.302944345	0.038053085
CD9	0.497915409	0.008004645	1.10342E-05
CD93	0.777630723	0.637056513	0.802452613
CD99	0.864493563	0.159816389	0.03724941
CD99L2	0.831814905	0.002093333	0.000197765
CDKN1A	0.696366743	0.488693315	0.689711797
CEBPB	0.372526739	0.064129821	0.205170016
CHI3L1	0.80580144	0.055087811	0.026946102
CHIT1	0.67192636	0.059574459	0.0533301
CHMP2A	0.939086234	0.929652788	0.828761677
CHMP4B	0.047672001	0.025257854	0.743435328
CIITA	0.342094794	0.218628421	0.713230681
CISH	0.811550341	0.000822292	3.5338E-06
CLEC4E	0.756788564	0.65459305	0.857031993
COL1A1	0.711914623	0.045711703	0.029841859
COX1	0.764817027	0.423539867	0.50741808
COX2	0.795200348	0.733165486	0.914343412
COX3	0.826536571	0.98986873	0.759111716
COX4I1	0.673903106	0.215345969	0.279833293
CSF1R	0.872617377	0.21148877	0.150262652
CTSB	0.571028613	0.814415674	0.289158288
CTSD	0.312322092	0.82243155	0.10237636
CTSL	0.77149409	0.803244843	0.956451897
CXCL16	0.777155721	0.29799706	0.317078323
CXCL2	0.784656154	0.014408693	0.006756789
CXCL3	0.264966203	0.574396776	0.031532729

CXCL5	0.612868017	0.190670875	0.016654668
CXCL8	0.006788892	0.332120738	1.38049E-06
CXCR2	0.709149552	0.62716764	0.262521295
CXCR4	0.683006293	0.039706761	0.029540643
CXCR5	0.004136043	0.000423297	0.304607018
CYBB	0.493332077	0.765737724	0.193514848
DDIT3	0.583639983	0.07118973	0.002134438
EIF2AK3	0.606234139	0.940199127	0.56183459
FCGR1A	0.76285316	0.853142831	0.877560638
FCGR3A	0.304988171	0.942566116	0.207342983
FN1	0.856873915	0.005183824	0.000543415
FUNDC1	0.855468696	0.764343027	0.536309303
GAA	0.259570547	0.779683899	0.262447752
GADD45A	0.494447233	0.69183472	0.70412228
GBA	0.037263882	0.104543363	0.542916647
GCLC	0.94691888	0.105101116	0.039857704
GCLM	0.199664599	0.429232919	0.516169039
GLUL	0.447959901	0.870393426	0.43077964
GPX1	0.547976259	0.002463096	0.001335869
GPX4	0.982600677	0.157989537	0.066542419
HLA-A	0.6950065	0.012042114	0.000124282
HLA-B	0.761183894	0.044256164	0.024018196
HLA-C	0.819944162	0.220233635	0.054735581
HLA-DPA1	0.638079121	0.477441163	0.118604993
HLA-DPB1	0.84663117	0.175806564	0.125307328
HLA-DRA	0.894155601	0.130779218	0.068391215
HLA-DRB1	0.887860338	0.059388141	0.02110789
HMGB1	0.004454434	0.00774248	0.808990784
HMOX1	0.700171308	0.755944634	0.921729279
HSPA1A	0.784498465	0.602844775	0.041927621
HSPA1B	0.960817331	0.58031348	0.138519487
HSPA5	0.27240316	0.026278243	0.137012021
ICAM2	0.810301854	0.96572152	0.797002005
IFIT1	0.094488376	0.097296462	0.98003919
IFIT2	0.697770183	0.664548813	0.28018526

IFIT3	0.224631986	0.374553769	0.661622764
IFNGR1	0.423861736	0.576107804	0.750282756
IKBKB	0.199506171	0.259081111	0.836673307
IL10	0.735840562	0.603373479	0.262130046
IL10RA	0.781189891	0.327034159	0.096706416
IL16	0.504588888	0.808581693	0.229798288
IL18	0.045506726	0.57101991	0.059882193
IL18BP	1.85055E-06	0.000922888	0.054026042
IL1B	0.050122388	0.490834478	0.000559684
IL1R1	0.976045932	0.10207319	0.036381492
IL21R	0.842256783	0.025204143	0.001357402
IL27RA	0.503922217	0.094946794	0.185728976
IL2RG	0.730756043	0.149566756	0.1467696
IL4I1	0.455358927	0.048192556	0.105041252
IL4R	0.004462735	0.001231739	0.626711815
IL6R	0.904949185	0.490501167	0.453120348
IL7	0.792097828	0.167975871	0.157189278
IL7R	0.974913926	0.001139279	2.12731E-05
IRAK1	0.8514346	0.289946552	0.250714999
IRAK3	0.93072102	0.778679499	0.798903937
IRAK4	0.964333934	0.344047744	0.243742609
IRF1	0.059403698	0.330030928	0.229921185
IRF2	0.661471842	0.12039468	0.008781977
IRF3	0.265248693	0.973646102	0.155685901
IRF5	0.010660512	0.025230925	3.43416E-05
IRF7	0.316183884	0.553571916	0.997545967
IRF8	0.655017569	0.397899953	0.154747317
IRF9	0.707136813	0.313213573	0.564235605
ITGAM	0.570866272	0.528078089	0.043015647
ITGB2	0.031486965	0.949339868	0.068427095
JUNB	0.883340839	0.123701921	0.065596772
LAMP1	0.177340952	0.67347145	0.219885802
LAMP2	0.767288672	0.023617663	0.000730363
LDLR	0.405205633	0.742991048	0.125549519
LGALS3	0.002144686	0.000158387	1.44618E-19

LIPA	0.514727046	0.901670047	0.484947756
LRP1	0.176921101	0.111491851	0.749678429
LYZ	0.763459661	0.574858766	0.730925223
MACROH2A1	0.154047616	0.20535281	0.000379426
MAFB	0.983155664	0.882855193	0.867397719
MAP1LC3A	0.950097828	0.339852558	0.189954915
MAP1LC3B	0.002885306	0.931879555	5.72968E-05
MAP1LC3B2	0.990518616	0.614233003	0.614233003
MAP1LC3C	0.942570305	0.734710411	0.727567729
MAPK1	0.290237001	0.517545026	0.024969375
MARCO	0.460186143	0.536521929	0.873263525
MCOLN1	0.789000988	0.481342526	0.199442734
MERTK	0.887525371	0.829943589	0.638447281
MMP14	2.20673E-06	1.35998E-07	0.480040355
MMP19	0.16829196	0.075189657	0.596104408
MMP2	0.034671338	0.174442275	0.318488493
MMP24OS	0.30571267	0.011063631	0.049270604
MMP25	1.2759E-05	0.000242254	0.346668344
MMP7	0.908192982	0.280591311	0.114124786
MMP8	0.498521458	0.127136836	0.003811837
MMP9	0.098235921	0.00517452	0.130693428
MRC1 (CD206)	0.934010058	0.048120369	0.012305
MSR1	0.421448298	0.038899821	0.095340688
MYD88	0.886475703	0.534096895	0.312099716
ND1	0.571619884	0.063374138	0.087928688
ND2	0.744483557	0.058646972	0.038628273
ND3	0.274425018	3.35107E-06	2.61483E-06
ND4	0.758578885	0.090171816	0.066576584
ND4L	0.582210577	0.855850588	0.626432215
ND5	0.245741261	0.023685536	0.145337742
ND6	0.270979798	0.265685866	0.987960577
NFKB1	0.501209741	0.034730965	0.059078016
NFKB2	0.052188505	6.26406E-06	0.000689133
NFKBIA	0.732264215	0.897256404	0.536288398
NLRP3	0.676311929	0.606304458	0.898149381

NOTCH2	0.052287808	0.545577239	0.077295066
NQO1	7.01785E-06	0.515207435	4.15879E-07
NRP1	0.919188181	0.6044145	0.581830884
NRP2	0.058046097	0.011001364	0.393117532
OAS1	0.887217964	0.225456006	0.159338306
OAS2	0.714360657	0.808668084	0.422607717
OAS3	0.913679713	0.813422219	0.649443588
PARP1	0.55038615	0.070912805	0.001556979
PIK3AP1	0.83329915	0.464067532	0.491567084
PLIN2	0.837028762	0.02666681	7.80614E-06
PPARA	0.200977489	0.571002936	0.016138784
PPARD	0.291440051	4.69611E-05	6.89129E-05
PPARG	0.874874758	0.735696618	0.81269537
PYCARD	0.103089317	0.216124983	0.602329819
RAB7A	0.033786923	0.503877195	0.000236961
RAB7B	0.907691398	0.191314153	0.060134798
RAC1	0.753946035	0.236837694	0.251104093
RAD51	0.000111888	0.933831465	7.27379E-07
RB1CC1	0.494634797	0.69446791	0.16057598
RHOA	0.568202358	0.039326093	0.049196057
S100A11	0.205874132	0.589956094	0.337276715
S100A6	0.56190613	0.001903322	0.000857487
S100A8	0.834810671	0.130192366	0.084882605
S100A9	0.395151403	0.809051732	0.421320654
SERPINA1	0.123607074	0.106788376	3.23969E-05
SERPINB1	0.953395945	0.149837839	0.049422242
SERPINB2	0.174438526	0.542118791	0.009314708
SERPINB6	0.191691607	0.104141946	0.000121621
SERPINB8	0.820309954	0.944862998	0.835578987
SERPINB9	0.631951382	0.01033896	0.006355434
SIRPA	0.007115226	0.004887913	0.871917133
SLAMF8	0.014418923	0.011702288	0.923756399
SNAP29	0.757230202	0.513235747	0.207393256
SOCS3	0.509401438	0.219020017	0.464842031
SOD1	0.602996268	0.115093933	0.166931636

SOD2	0.322736497	0.901004095	0.253048371
SPARC	0.896313073	0.11060877	0.052600875
SPP1 (OPN)	0.669750005	0.417299193	0.101589748
SQSTM1	0.00036306	0.517014298	0.000115797
SREBF1	0.585165994	0.908467726	0.569009673
SREBF2	0.13862909	0.620586479	0.193163409
STAT1	0.585785014	0.208761837	0.348644076
STAT2	0.043186479	0.070129446	0.778108022
STAT3	0.338604819	0.339832559	0.996519183
STAT4	0.52378025	0.92346077	0.470651869
STAT5A	0.173653822	0.757116635	0.027827225
STAT5B	0.454018225	0.262224883	0.014630573
STAT6	0.180648632	0.400083857	0.51137366
STX17	0.586405513	0.166206978	0.012633647
TFAM	0.271249294	0.810717675	0.079382981
TGFBI	0.035867512	0.214620255	0.008678165
TLR1	0.186412313	0.840986356	0.046261564
TLR2	0.931670985	0.37638321	0.200035179
TLR3	0.856352036	0.705837267	0.797134841
TLR4	0.871526315	0.412024486	0.383935312
TLR5	0.97371054	0.480818342	0.376934108
TLR6	0.857653976	0.817134732	0.946731503
TLR7	0.540939733	0.782638805	0.657365968
TLR8	0.025002985	0.323640948	0.098609139
TNF	0.289206993	0.360547823	0.841271671
TP53	0.250773909	0.00032696	0.001449258
TRAF6	0.196026168	0.600682167	0.312517702
TREM2	0.925281645	0.897597435	0.963182738
TXNRD1	0.022336705	0.123894768	0.323738048
ULK1	0.129373731	0.127612206	0.995415621
ULK2	0.379363608	0.30101526	0.012095385
VAMP8	0.459902144	0.104390381	0.24346044
VPS4A	0.33787987	0.780984539	0.104869964
VPS4B	0.825658163	0.518221854	0.255870825
WDR1	0.926507347	0.818226652	0.670301426

XBP1	0.590358349	0.214148738	0.355823769
XRCC1	0.532232255	0.385223327	0.052718515

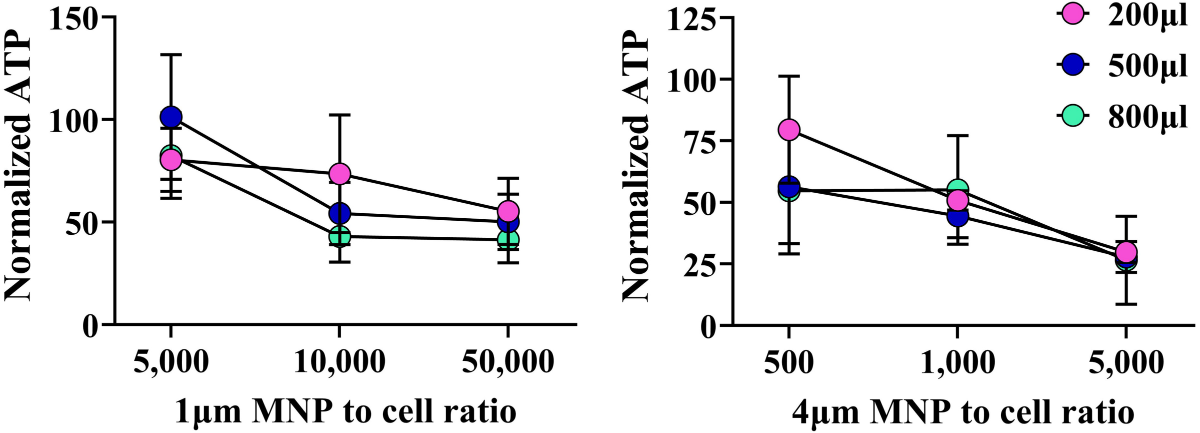

Normalized ATP
150
100
50
0
5,000
10,000
50,000
1µm MNP to cell ratio
Normalized ATP
125
100
75
50
25
0
500
1,000
5,000
4µm MNP to cell ratio
200µl
500µl
800µl

4μm

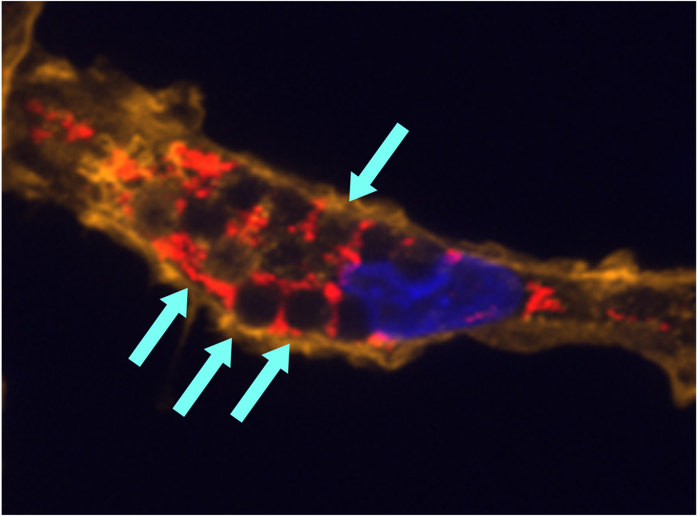

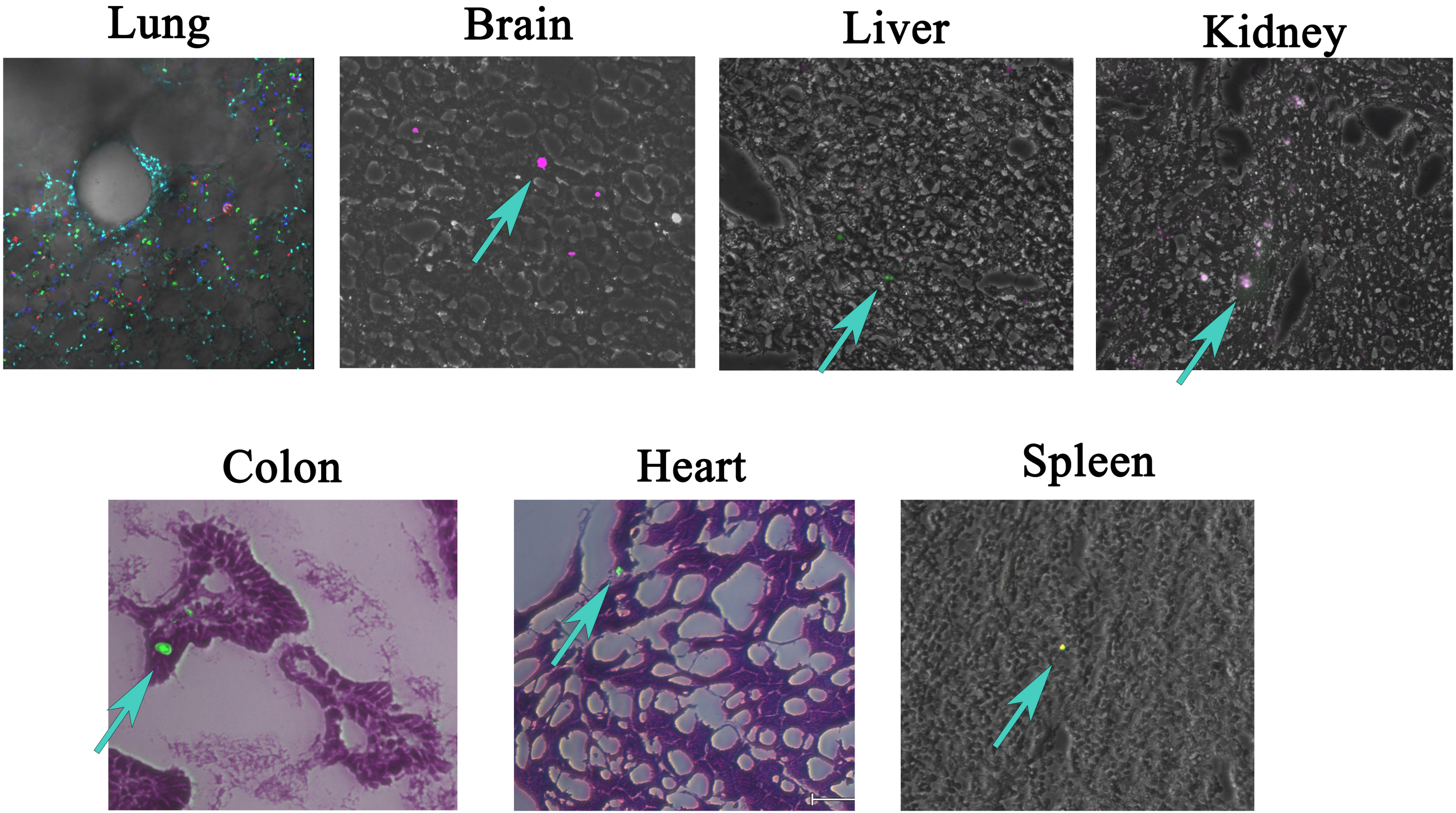
Lung
Brain
Liver
Kidney
Colon
Heart
Spleen
